# Lenalidomide maintenance versus observation for patients with newly diagnosed multiple myeloma (Myeloma XI): a multicentre, open-label, randomised, phase 3 trial

**DOI:** 10.1016/S1470-2045(18)30687-9

**Published:** 2019-01

**Authors:** Graham H Jackson, Faith E Davies, Charlotte Pawlyn, David A Cairns, Alina Striha, Corinne Collett, Anna Hockaday, John R Jones, Bhuvan Kishore, Mamta Garg, Cathy D Williams, Kamaraj Karunanithi, Jindriska Lindsay, Matthew W Jenner, Gordon Cook, Nigel H Russell, Martin F Kaiser, Mark T Drayson, Roger G Owen, Walter M Gregory, Gareth J Morgan

**Affiliations:** a Northern Institute for Cancer Research, Newcastle University, Newcastle upon Tyne, UK; b The Myeloma Institute, University of Arkansas for Medical Sciences, Little Rock, AR, USA; c The Institute of Cancer Research, London, UK; d The Royal Marsden Hospital NHS Foundation Trust, London, UK; e Clinical Trials Research Unit, Leeds Institute of Clinical Trials Research, University of Leeds, Leeds, UK; f Section of Experimental Haematology, Leeds Institute of Cancer and Pathology, University of Leeds, Leeds, UK; g Heart of England NHS Foundation Trust, Birmingham, UK; h Leicester Royal Infirmary, Leicester, UK; i Centre for Clinical Haematology, Nottingham University Hospital, Nottingham, UK; j University Hospital of North Midlands, Stoke-on-Trent, UK; k East Kent Hospitals University NHS Foundation Trust, Canterbury, UK; l University Hospital Southampton NHS Foundation Trust, Southampton, UK; m Clinical Immunology Service, Institute of Immunology and Immunotherapy, University of Birmingham, Birmingham, UK; n Haematological Malignancy Diagnostic Service, St James's University Hospital, Leeds, UK

## Abstract

**Background:**

Patients with multiple myeloma treated with lenalidomide maintenance therapy have improved progression-free survival, primarily following autologous stem-cell transplantation. A beneficial effect of lenalidomide maintenance therapy on overall survival in this setting has been inconsistent between individual studies. Minimal data are available on the effect of maintenance lenalidomide in more aggressive disease states, such as patients with cytogenetic high-risk disease or patients ineligible for transplantation. We aimed to assess lenalidomide maintenance versus observation in patients with newly diagnosed multiple myeloma, including cytogenetic risk and transplantation status subgroup analyses.

**Methods:**

The Myeloma XI trial was an open-label, randomised, phase 3, adaptive design trial with three randomisation stages done at 110 National Health Service hospitals in England, Wales, and Scotland. There were three potential randomisations in the study: induction treatment (allocation by transplantation eligibility status); intensification treatment (allocation by response to induction therapy); and maintenance treatment. Here, we report the results of the randomisation to maintenance treatment. Eligible patients for maintenance randomisation were aged 18 years or older and had symptomatic or non-secretory multiple myeloma, had completed their assigned induction therapy as per protocol and had achieved at least a minimal response to protocol treatment, including lenalidomide. Patients were randomly assigned (1:1 from Jan 13, 2011, to Jun 27, 2013, and 2:1 from Jun 28, 2013, to Aug 11, 2017) to lenalidomide maintenance (10 mg orally on days 1–21 of a 28-day cycle) or observation, and stratified by allocated induction and intensification treatment, and centre. The co-primary endpoints were progression-free survival and overall survival, analysed by intention to treat. Safety analysis was per protocol. This study is registered with the ISRCTN registry, number ISRCTN49407852, and clinicaltrialsregister.eu, number 2009-010956-93, and has completed recruitment.

**Findings:**

Between Jan 13, 2011, and Aug 11, 2017, 1917 patients were accrued to the maintenance treatment randomisation of the trial. 1137 patients were assigned to lenalidomide maintenance and 834 patients to observation. After a median follow-up of 31 months (IQR 18–50), median progression-free survival was 39 months (95% CI 36–42) with lenalidomide and 20 months (18–22) with observation (hazard ratio [HR] 0·46 [95% CI 0·41–0·53]; p<0·0001), and 3-year overall survival was 78·6% (95% Cl 75·6–81·6) in the lenalidomide group and 75·8% (72·4–79·2) in the observation group (HR 0·87 [95% CI 0·73–1·05]; p=0·15). Progression-free survival was improved with lenalidomide compared with observation across all prespecified subgroups. On prespecified subgroup analyses by transplantation status, 3-year overall survival in transplantation-eligible patients was 87·5% (95% Cl 84·3–90·7) in the lenalidomide group and 80·2% (76·0–84·4) in the observation group (HR 0·69 [95% CI 0·52–0·93]; p=0·014), and in transplantation-ineligible patients it was 66·8% (61·6–72·1) in the lenalidomide group and 69·8% (64·4–75·2) in the observation group (1·02 [0·80–1·29]; p=0·88). By cytogenetic risk group, in standard-risk patients, 3-year overall survival was 86·4% (95% CI 80·0–90·9) in the lenalidomide group compared with 81·3% (74·2–86·7) in the observation group, and in high-risk patients, it was 74.9% (65·8–81·9) in the lenalidomide group compared with 63·7% (52·8–72·7) in the observation group; and in ultra-high-risk patients it was 62·9% (46·0–75·8) compared with 43·5% (22·2–63·1). Since these subgroup analyses results were not powered they should be interpreted with caution. The most common grade 3 or 4 adverse events for patients taking lenalidomide were haematological, including neutropenia (362 [33%] patients), thrombocytopenia (72 [7%] patients), and anaemia (42 [4%] patients). Serious adverse events were reported in 494 (45%) of 1097 patients receiving lenalidomide compared with 150 (17%) of 874 patients on observation. The most common serious adverse events were infections in both the lenalidomide group and the observation group. 460 deaths occurred during maintenance treatment, 234 (21%) in the lenalidomide group and 226 (27%) in the observation group, and no deaths in the lenalidomide group were deemed treatment related.

**Interpretation:**

Maintenance therapy with lenalidomide significantly improved progression-free survival in patients with newly diagnosed multiple myeloma compared with observation, but did not improve overall survival in the intention-to-treat analysis of the whole trial population. The manageable safety profile of this drug and the encouraging results in subgroup analyses of patients across all cytogenetic risk groups support further investigation of maintenance lenalidomide in this setting.

**Funding:**

Cancer Research UK, Celgene, Amgen, Merck, and Myeloma UK.

## Introduction

Most patients with newly diagnosed multiple myeloma have achieved survival benefits from the therapeutic advances made during the past decade, but patients with high-risk subsets of disease have benefited less.^1^ High-risk disease can be identified at presentation by the cytogenetic lesions t(4;14), t(14;16), t(14;20), del(17p), and gain(1q), which are good markers to predict early relapse. In patients with high-risk disease, relapse is the result of the persistence of residual disease,^2^, ^3^ even at very low levels, and is associated with clonal evolution and progressive immune dysfunction.^4^, ^5^ These biological features can impair the activity of subsequent lines of therapy; therefore, finding strategies that could prevent relapse in this group would be a considerable step forward to improve the outcomes of these patients.^6^

Control or elimination of residual disease clones might be achieved by maintenance therapy, and several strategies have been assessed in this setting, with lenalidomide being the most promising because of its mode of action and tolerability.^7^, ^8^, ^9^ Our preceding study, the Medical Research Council Myeloma IX trial,^10^ compared the use of the immunomodulatory agent thalidomide as maintenance therapy with observation in patients with newly diagnosed multiple myeloma. Treatment with thalidomide improved progression-free survival in these patients, but there was no improvement in overall survival. Importantly, in patients with cytogenetic high-risk disease, thalidomide did not improve progression-free survival or overall survival. Toxicity (including peripheral neuropathy, lethargy, and constipation) with thalidomide restricted therapy completion and might have affected the overall survival outcomes in this setting. Our findings were consistent with the results of other contemporaneous thalidomide maintenance studies.^11^, ^12^ The Myeloma XI study was therefore designed to assess whether the newer, better tolerated, immunomodulatory agent lenalidomide could overcome the limitations of thalidomide as maintenance therapy in this setting and improve progression-free survival and overall survival in patients with newly diagnosed multiple myeloma.

**Research in context**

**Evidence before this study**

A search of PubMed for clinical trial reports published in English before May, 2010, using the terms “lenalidomide” and “myeloma” and “maintenance” revealed no published randomised studies of lenalidomide maintenance in patients with newly diagnosed myeloma.

**Added value of this study**

To our knowledge, the Myeloma XI trial recruited more patients with newly diagnosed multiple myeloma than any previous interventional study in myeloma. We showed a significant benefit of lenalidomide maintenance therapy in terms of progression-free survival, which was consistent across both patients eligible for transplantation and those who were ineligible, as well as patients across all cytogenetic risk groups, even those with high-risk disease. The primary endpoint analysis of overall survival was not met. However, a preplanned subgroup analysis indicates an overall survival benefit in transplantation-eligible patients across all cytogenetic risk groups, even those with high-risk disease, when treated with lenalidomide maintenance therapy after transplantation.

**Implications of all the available evidence**

The results of the Myeloma XI trial contribute to a body of evidence that suggests that the use of lenalidomide as maintenance therapy should be considered for patients with newly diagnosed multiple myeloma, of all cytogenetic risk groups, after autologous stem-cell transplantation. With the addition of these new data from the Myeloma XI trial, a meta-analysis of all published trials of lenalidomide maintenance after autologous stem-cell transplantation, including 3179 patients, confirmed the overall survival benefit of lenalidomide maintenance therapy compared with observation in this setting (hazard ratio 0·72 [95% CI 0·56–0·91]). Future studies examining combinations of agents with lenalidomide are needed to further improve outcomes for high-risk patients with multiple myeloma. In transplantation-ineligible patients, novel approaches to improve overall survival are warranted.

Binding of lenalidomide to the cereblon complex^13^ leads to the ubiquitination of substrates, including the transcription factors Ikaros and Aiolos, marking them for proteasomal degradation and resulting in decreased expression of interferon regulatory factor 4 (IRF4).^14^, ^15^ Lenalidomide has direct tumouricidal effects on myeloma cells and also triggers indirect immunomodulatory effects, including activation of natural killer and T cells, which might help with elimination of minimal residual disease in patients with multiple myeloma.^14^, ^15^, ^16^, ^17^ Whether or not these mechanisms can help to control residual clonal disease in patients with high-risk cytogenetic disease is unknown.

Three previous studies have shown that lenalidomide maintenance can delay disease progression after autologous stem-cell transplantation in patients with multiple myeloma.^7^, ^8^, ^9^ Overall survival outcomes were inconsistent between these studies, none of which was appropriately powered to make robust conclusions based on this endpoint. One study^8^ showed a clear overall survival benefit for those patients treated with maintenance lenalidomide compared with observation, whereas the other two studies^7^, ^9^ showed no significant benefit. However, a recently published meta-analysis^18^ including these trials showed that lenalidomide maintenance can improve overall survival compared with observation in this setting, and it is now approved for use by both the European Medicines Agency and US Food and Drug Administration. For patients with multiple myeloma who are ineligible for transplantation, continuous therapy with lenalidomide plus low-dose dexamethasone improved progression-free survival and overall survival compared with a combination of melphalan, prednisone, and thalidomide.^19^, ^20^ No previous studies of either transplantation-eligible or transplantation-ineligible patients had sufficient numbers to assess the effect of lenalidomide maintenance therapy in patients with multiple myeloma and high-risk cytogenetics.

Here, we aimed to assess the effect of lenalidomide maintenance on the survival of patients with newly diagnosed multiple myeloma, including preplanned subgroup analyses by cytogenetic risk group and transplantation status.

## Methods

### Study design and participants

**Figure 1:**
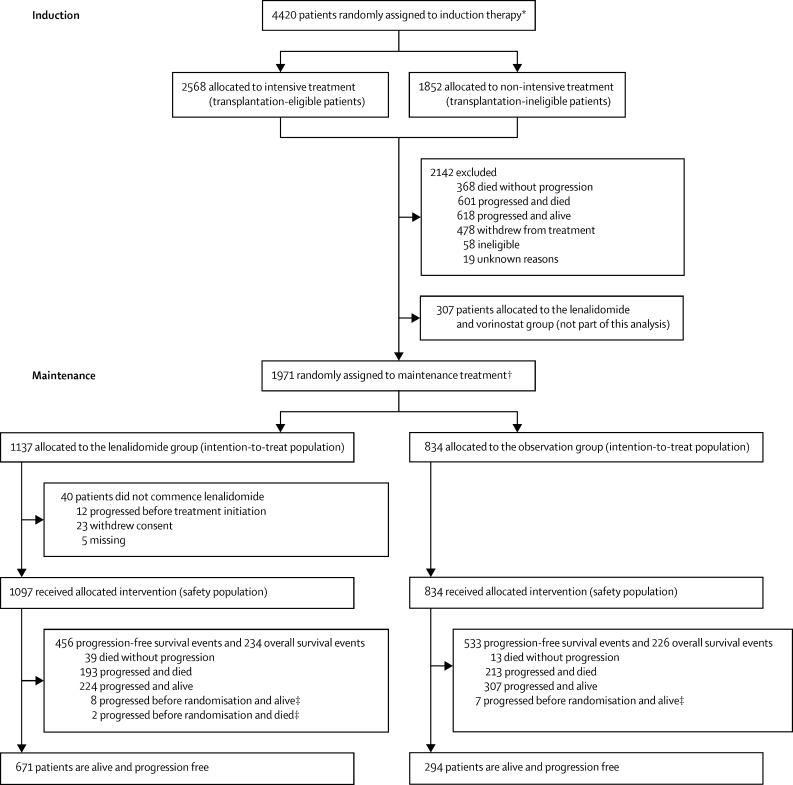
Trial profile *Randomisation occurred between May 26, 2010, and April 20, 2016. †Randomisation occurred between Jan 13, 2011, and Aug 11, 2017. ‡Censored for progression-free survival analysis.

The Myeloma XI was a phase 3, open-label, randomised, adaptive design trial with three randomisation stages (figure 1). There were three potential randomisations in the study: at trial entry for all patients to allocate induction treatment separately for those considered eligible or ineligible for transplantation; after induction treatment for those patients with a suboptimal response to treatment (minimal or partial response) to allocate induction intensification; and at the completion of induction and intensification or autologous stem-cell transplantation (where applicable) to allocate maintenance treatment. This report is concerned with the results of the randomisation to maintenance treatment. Results of the induction intensification randomisations will be presented elsewhere. The trial was done at 110 National Health Service hospitals in England, Wales, and Scotland (appendix p 2).

The full study protocol including the inclusion criteria for each randomisation is available in the appendix (p 34). Patients aged at least 18 years and who had symptomatic multiple myeloma or non-secretory multiple myeloma based on bone marrow clonal plasma cells, organ or tissue impairment considered by the clinician to be myeloma related, or paraprotein (M-protein) in serum or urine were eligible for the initial randomisation. Exclusion criteria for the initial randomisation included previous or concurrent malignancies, including myelodysplastic syndromes; previous treatment for myeloma (except local radiotherapy, bisphosphonates, and corticosteroids); grade 2 or worse peripheral neuropathy, acute renal failure (unresponsive to up to 72 h of rehydration, characterised by creatinine >500 μmol/L or urine output <400 mL per day, or requiring dialysis); and active or previous hepatitis C infection.

Patients who were young and fit to tolerate autologous stem-cell transplantation (transplantation eligible) entered the intensive treatment pathway. Older and less fit patients (transplantation ineligible) entered the non-intensive treatment pathway. Strict age limits were deliberately avoided so that fit, older patients could receive intensive therapy and undergo autologous stem-cell transplantation. However, generally, patients aged 60 years or younger entered the intensive (younger, fitter) pathway; those aged 70 years or older entered the non-intensive (older, less fit) pathway; and those aged 61–69 years were eligible for either intensive or non-intensive therapy. The decision of treatment pathway was made on an individual patient basis, taking into account Eastern Cooperative Oncology Group performance status, clinician judgment, and patient preference.

For the maintenance therapy randomisation, eligible patients were those who completed their assigned induction therapy according to the protocol (a minimum of four cycles of cyclophosphamide, thalidomide, and dexamethasone [CTD]; cyclophosphamide, lenalidomide, and dexamethasone [CRD]; or carfilzomib, cyclophosphamide, lenalidomide, and dexamethasone [KCRD] in the intensive pathway, or a minimum of six cycles of attenuated CTD or attenuated CRD in the non-intensive pathway), and had achieved at least a minimal response and received at least 100 mg/m^2^ melphalan if assigned to intensive treatment.

The study was approved by the national ethics review board (National Research Ethics Service, London, UK), institutional review boards of the participating centres, and the competent regulatory authority (Medicines and Healthcare Products Regulatory Agency, London, UK), and was undertaken according to the Declaration of Helsinki and the principles of Good Clinical Practice as espoused in the Medicines for Human Use (Clinical Trials) Regulations. All patients provided written informed consent. The study is closed for accrual, but follow-up continues for planned long-term analysis.

### Randomisation and masking

Patients considered eligible for transplantation at trial entry were randomly assigned (1:1) to induction treatment with either CTD or CRD. A computer-generated minimisation algorithm was used to avoid chance imbalances in six variables measured at trial entry: β_2_ microglobulin (<3·5 mg/L *vs* 3·5–<5·5 mg/L *vs* ≥5·5 mg/L *vs* or unknown), haemoglobin (<115 g/L *vs* ≥115 g/L for men; <95 g/L *vs* ≥95 g/L for women), corrected serum calcium (<2·6 mmol/L *vs* ≥2·6 mmol/L), serum creatinine (<140 μmol/L *vs* ≥140 μmol/L), platelets (<150 × 10^9^ cells per L *vs* ≥150 × 10^9^ cells per L), and centre (each centre is listed in the appendix pp 2–4). Following a protocol amendment on June 28, 2013, and after the accrual of 1512 patients, patients considered eligible for transplantation were randomly assigned (1:1:2) to CTD, CRD, or KCRD. A similar minimisation algorithm was used to avoid chance imbalances in the six variables measured at trial entry. These changes were made to add research questions to this adaptive design study. Patients considered ineligible for transplantation at trial entry were randomly assigned (1:1) to induction with either attenuated CTD or attenuated CRD. A similar minimisation algorithm was used to avoid chance imbalances in the six variables measured at trial entry.

Patients with a suboptimal response to induction treatment were randomly assigned (1:1) to cyclophosphamide, bortezomib and dexamethasone (CVD) or no CVD. A minimisation algorithm was used to avoid chance imbalances in three variables: allocated induction treatment (CTD *vs* CRD *vs* attenuated CTD *vs* attenuated CRD), response to induction treatment (minimal response or partial response), and centre. Patients allocated to KCRD induction treatment were ineligible for this randomisation.

Patients completing induction and intensification treatment (where applicable) and eligible were randomly assigned (1:1) to lenalidomide maintenance or observation. A minimisation algorithm was used to avoid chance imbalances in three variables: allocated induction treatment (CTD *vs* CRD *vs* attenuated CTD *vs* attenuated CRD), allocated intensification treatment (no CVD *vs* CVD *vs* not randomised at intensification randomisation), and centre. Following a protocol amendment on Sept 14, 2011, and after accrual of 442 patients under protocol versions 2.0 to 4.0, patients were randomly assigned (1:1:1) to lenalidomide, lenalidomide plus vorinostat, or observation. A similar minimisation algorithm was used to avoid chance imbalances in the same three variables with the same categories. Following a protocol amendment on June 28, 2013, and after accrual of 615 further patients under protocol version 5.0, patients were randomly assigned (2:1) to lenalidomide or observation. A similar minimisation algorithm was used to avoid chance imbalances in the same three variables with the same categories but with the addition of KCRD to the induction treatment options. These changes were made to add and remove research questions in this adaptive design study.

All randomisations were done at the Clinical Trials Research Unit (Leeds, UK) by authorised members of staff with a centralised automated 24 h telephone system according to a validated minimisation algorithm produced under the supervision of WMG. Because of the nature of the intervention, the study was open label and the allocated treatment was not masked from study investigators or patients. The funders remained masked to treatment results until data cutoff for analysis.

### Procedures

The dose, schedule, and route of administration of each drug included in the induction and consolidation regimens are in the protocol (appendix p 34). Briefly, in transplantation-eligible patients, induction therapy with CTD, CRD, or KCRD continued for at least four cycles in the absence of progressive disease, until maximum response or intolerance was observed. In transplantation-ineligible patients, attenuated CTD or attenuated CRD continued for at least six cycles in the absence of progressive disease, until maximum response or intolerance was observed. Attenuated versions of induction included lower doses of dexamethasone and a lower starting dose of thalidomide. For all patients, bisphosphonates were recommended until progressive disease and thromboprophylaxis was recommended for at least the first 3 months of treatment as per IMWG recommendations. Growth factor support and prophylaxis for pneumonia, varicella, fungal infection, and tumour lysis syndrome were allowed as per local practice.

Transplantation-eligible patients receiving KCRD proceeded to high-dose melphalan and autologous stem-cell transplantation. Patients receiving immunomodulatory-based triplets (CTD *vs* CRD) followed a response-adapted approach: those with complete response or very good partial response (assessed according to International Myeloma Working Group [IMWG] criteria) proceeded to autologous stem-cell transplantation in the transplantation-eligible pathway, whereas transplantation-ineligible patients proceeded directly to maintenance randomisation. For maintenance therapy, 100 days after autologous stem-cell transplantation or once a maximum response was achieved for transplantation-ineligible patients, patients initially received lenalidomide 25 mg per day (orally on days 1–21 of each 28-day cycle) or were observed without lenalidomide therapy. Following a protocol amendment on Sept 14, 2011, and after accrual of 442 patients, patients were allocated (1:1:1) to receive lenalidomide 10 mg per day (orally on days 1–21 of each 28-day cycle), lenalidomide 10 mg per day (orally on days 1–21 of each 28-day cycle) plus vorinostat 300 mg per day (orally on days 1–7 and 15–21 of each 28-day cycle), or observation. After accrual of 615 more patients, a further protocol amendment on June 28, 2013, allocated patients to receive lenalidomide 10 mg per day (orally on days 1–21 of each 28-day cycle) or observation in a 2:1 ratio, and the lenalidomide plus vorinostat group was discontinued. This change was proposed by the Myeloma XI Trial Management Group and approved by the Myeloma XI Data Monitoring and Ethics Committee (DMEC) on June 24, 2011. The change in dose was based on emerging results from previous studies using 10 mg lenalidomide weighted against the potential for late toxicity (increased secondary primary malignancy), which was being reported in other trials at that time. Maintenance treatment continued until progressive disease in the absence of toxicity. Here, we report outcomes for patients receiving lenalidomide alone versus observation. Patients receiving lenalidomide in combination with vorinostat will be reported elsewhere when the primary endpoint has been met in that group.

Response and disease progression were assessed on the basis of IMWG Uniform Response criteria^21^, ^22^ and reviewed centrally by an expert panel masked to treatment allocation. Adverse events were graded according to the US National Cancer Institute Common Terminology Criteria for Adverse Events (NCI CTCAE), version 4.0. Adverse reactions were assessed at the start of each treatment cycle in patients receiving lenalidomide. Serious adverse events were reported for all patients from the date of randomisation until 30 days after the date of disease progression except in the case of serious adverse reactions or second primary malignancies, which were collected for the duration of the trial. Second primary malignancies were reported as serious adverse events for the duration of the study (ie, until death for each patient or when the study closes, whichever was earlier). The definition of secondary primary malignancies excluded non-melanoma skin cancers such as squamous and basal cell carcinomas of the skin. Paraprotein, serum-free light-chain analysis, and urinary light chain excretion were assessed at least every 2 months for the first 2 years and then at least every 3 months until disease progression.

Lenalidomide dose reductions due to adverse reactions were allowed. Treatment was discontinued for grade 3 or 4 neutropenia and for a platelet count of less than 30 × 10^9^/L. On recovery, treatment was restarted at the previous dose, with the addition of granulocyte colony-stimulating factor in the case of grade 3 neutropenia with fever or grade 4 neutropenia. If neutropenia or thrombocytopenia occurred a second time, then the dose was reduced by one dose level as stipulated in the protocol (eg, from 10 mg daily to 5 mg daily). Full details of the dose reduction schedules are shown in the protocol (appendix p 34).

Cytogenetic risk profiling was performed by use of multiplex ligation-dependent probe amplification and quantitative real-time PCR using DNA and RNA respectively, which was extracted from CD138-selected plasma cells from bone marrow biopsy samples taken before treatment initiation. Quantitative real-time PCR was used to assess the expression of translocation gene partners including t(4;14):*MMSET, FGFR3*, t(14;16):*MAF*, and t(14;20):*MAFB*. Multiplex ligation-dependent probe amplification was used to assess copy number aberrations by including probe sets at sites of the commonly deleted or amplified regions in myeloma (eg, at genes *CKS1B* on 1q21.3 and *TP53* on 17p13). These techniques are validated and provide equivalent results to interphase fluorescence in-situ hybridisation.^23^, ^24^, ^25^ Patients were classified into three cytogenetic risk groups for the preplanned analysis of outcomes: standard risk (no adverse cytogenetic abnormalities), high risk (one adverse cytogenetic abnormality), or ultra-high risk (two or more adverse cytogenetic abnormalities). Adverse cytogenetic abnormalities were defined as gain(1q), t(4;14), t(14;16), t(14;20), or del(17p).^2^, ^3^ In a post-hoc analysis, an alternative cytogenetic high-risk classification was used, including patients with t(4;14), del(17p), or t(4;14) and del(17p).^7^

### Outcomes

The co-primary endpoints of the maintenance evaluation of the trial were progression-free survival and overall survival. Progression-free survival was defined as the time from maintenance randomisation to progressive disease or death from any cause. Overall survival was defined as the time from maintenance randomisation to death from any cause or last follow-up.

Secondary endpoints were progression-free survival 2, defined as the time from maintenance randomisation to the date of second progressive disease, start of third antimyeloma treatment, or death from any cause; the time to improved response; and toxicity. Exploratory analyses of progression-free survival, overall survival, and response by cytogenetic risk group in the overall population and by cytogenetic risk group were prespecified by protocol and by induction/intensification treatment were prespecified in the statistical analysis plan within each pathway.

### Statistical analysis

The data cutoff date for this analysis was Oct 23, 2017. We present here the results of the co-primary endpoints, some secondary endpoints (progression-free survival 2 and toxicity), and prespecified subgroup analysis for the maintenance randomisation of this trial. Response (secondary endpoint) and other exploratory endpoints will be reported elsewhere. The hypothesis of the maintenance randomisation was that lenalidomide treatment could improve progression-free survival and overall survival compared with observation in adult patients with multiple myeloma. For progression-free survival, the trial was designed to demonstrate a 6·7-month increase in median progression-free survival in the lenalidomide group (median 26·7 months) compared with the observation group (median 20·0 months, hazard ratio [HR] 0·75) when 509 progression-free survival events had been observed. For overall survival, it was designed to demonstrate a 10% increase in 5-year overall survival in the lenalidomide group (60% at 5 years) compared with the observation group (50% at 5 years, HR 0·74) when 458 overall survival events had been observed. Each of these calculations assumed the time to event was exponentially distributed and that recruitment would last 3·25 years with 4 years of further follow-up, a two-sided 5% significance level, and 90% power. A minimum recruitment target of 1013 patients randomly assigned to (1:1) lenalidomide and observation was specified, allowing for a 2% dropout. Efficacy analyses were done by intention to treat, including all patients randomly assigned to either lenalidomide alone or observation. The safety population included all patients who received at least one dose of maintenance therapy or those were assigned to observation. Patients randomly assigned during a transient period of the trial to the combination of lenalidomide and vorinostat (n=307), as per the protocol modification on Sept 14, 2011, were excluded from this analysis and will be reported elsewhere.

For the co-primary endpoints, we estimated summaries of time to event per treatment group using the Kaplan-Meier method. We made comparisons between the allocated groups using the Cox proportional hazards model stratified by the minimisation stratification factors, excluding centre, and to estimate HRs and 95% CIs. We used similar methods to assess the secondary outcome of progression-free survival 2. We also did sensitivity analyses for progression-free survival using similar models in which those participants identified to have progressed in advance of maintenance randomisation were defined as having an event at the time of maintenance randomisation. Subgroup analysis was prespecified for the presence or absence of individual adverse cytogenetic abnormalities, cytogenetic risk status, and induction and consolidation treatment (CVD intensification and transplantation eligibility). We did a likelihood ratio test for heterogeneity of treatment effect using Cox models identical to those used for the main analysis, with the inclusion of terms for the subgroup in question and the appropriate interaction term. The reported test for heterogeneity for subgroup analysis corresponds to a one degree of freedom test for two category subgroups and a two degrees of freedom test for three category subgroups.

We summarised toxicity, in terms of adverse events, descriptively. We estimated cumulative incidence function curves for time to second primary malignancies by non-parametric maximum likelihood estimation. We used Fine and Gray competing risks regression to compare the hazard of second primary malignancies by allocated treatment, adjusting for the minimisation stratification factors, with unrelated deaths specified as a competing risk. We calculated person-years on trial as the sum of all patients receiving at least one dose of study treatment of the time in years from randomisation to death or last date known to be alive. We calculated incidence with the number of events as the numerator and the number of person-years on trial as the denominator. We calculated 95% CIs for incidence using approximations to the Poisson distribution.

Post-hoc exploratory analyses undertaken were the effect on progression-free survival, overall survival, and progression-free survival 2 of the subgroups sex, age, disease stage according to the International Staging System, and response at start of maintenance; analysis of the effect of induction or intensification treatment and cytogenetic risk group on progression-free survival 2; analysis of the patients subsequently receiving lenalidomide in later lines of therapy; and a meta-analysis including the transplantation-eligible patients of this trial and those of previously published trials.

A formal interim analysis was prespecified in the study protocol for overall survival when at least 50% of required events had been observed (≥229 deaths). To ensure an overall significance level of 5% was maintained, we used the O'Brien and Fleming alpha-spending function with bounds specified at the time of the interim analysis related to the proportion of information accrued (interim analysis bound 0·94%, final analysis bound 4·7%). The bound for the interim analysis was advisory, with the decision to release results at the recommendation of the Independent Myeloma XI DMEC and the Independent Myeloma XI Trial Steering Committee. The interim analysis was done and presented to the DMEC on Sept 1, 2016, and the study continued without reporting the interim analysis. All reported p values are two sided and considered significant at an overall significance level of 5%.

We used SAS (version 9.4), Stata/IC, and R (version 3.2.3) for statistical analyses.

This study is registered with the ISRCTN registry, number ISRCTN49407852, and clinicaltrialsregister.eu, number 2009-010956-93.

### Role of the funding source

The funders of the study had no role in study design, data collection, data analysis, data interpretation, or writing of the report. All authors had full access to all the raw data in the study. The corresponding author had full access to all the data in the study and had final responsibility for the decision to submit for publication.

## Results

1971 patients were accrued to the maintenance randomisation between Jan 13, 2011, and Aug 11, 2017. 1137 patients were assigned to receive lenalidomide alone and 834 patients were assigned to observation (figure 1). Patient and disease characteristics were well balanced between groups (table 1).

**Table 1:** Baseline characteristics

			**Lenalidomide group (n=1137)**	**Observation group (n=834)**
Age, years			66 (59–72)	66 (59–72)
Age group				
	18–60 years		361 (32%)	251 (30%)
	61–70 years		416 (37%)	312 (37%)
	>70 years		360 (32%)	271 (32%)
Sex				
	Men		696 (61%)	527 (63%)
	Women		441 (39%)	307 (37%)
Ethnicity				
	White		1060 (93%)	773 (93%)
	Black (black Caribbean, black African, other)		26 (3%)	17 (2%)
	Asian (Indian, Pakistani, Bangladeshi, other)		18 (2%)	17 (2%)
	Other		10 (1%)	8 (1%)
	Unknown		23 (2%)	19 (2%)
Disease stage				
	I		327 (29%)	239 (29%)
	II		439 (39%)	349 (42%)
	III		291 (26%)	192 (23%)
	Unknown		80 (7%)	54 (6%)
Immunoglobulin subtype				
	IgG		699 (61%)	494 (59%)
	IgA		272 (24%)	219 (26%)
	IgM		7 (1%)	3 (<1%)
	IgD		12 (1%)	6 (1%)
	Light-chain only		137 (12%)	108 (13%)
	Non-secretor		9 (1%)	2 (<1%)
	Unknown		1 (<1%)	2 (<1%)
Creatinine, μmol/L			85 (71–103)	84 (69–105)
	Unknown		3 (<1%)	1 (<1%)
Lactate dehydrogenase, IU/L			262 (178–381)	271 (183–366)
	Unknown		251 (22%)	175 (21%)
Cytogenetic risk assessment available			447 (39%)	327 (39%)
Cytogenetic risk				
	Standard		228/447 (51%)	184/327 (56%)
	High risk*		166/447 (37%)	113/327 (35%)
	Ultra-high risk*		53/447 (12%)	30/327 (9%)
Transplantation eligibility and induction regimen†				
	Transplantation eligible		730 (64%)	518 (62%)
		CTD	236 (21%)	194 (23%)
		CRD	260 (23%)	207 (25%)
		KCRD	234 (21%)	117 (14%)
	Transplantation ineligible		407 (36%)	316 (38%)
	Attenuated CTD		194 (17%)	150 (18%)
	Attenuated CRD		213 (19%)	166 (20%)
CVD randomisation after minimal or partial response†				
	Allocated to CVD		79 (7%)	63 (8%)
	Allocated to no CVD		98 (9%)	78 (9%)
Received CVD after stable or progressive disease			16 (1%)	8 (1%)
Response at maintenance randomisation				
	Complete or very good partial response		945 (83%)	705 (85%)
	Partial or minimal response		172 (15%)	118 (14%)
	Stable or progressive disease		8 (1%)	6 (1%)
	Unable to assess		10 (1%)	1 (<1%)
	Unknown		2 (<1%)	4 (<1%)

Data are median (IQR), n (%), or n/N (%). CRD=cyclophosphamide, lenalidomide, and dexamethasone. CTD=cyclophosphamide, thalidomide, and dexamethasone. CVD=cyclophosphamide, bortezomib, and dexamethasone. KCRD=carfilzomib, cyclophosphamide, lenalidomide, and dexamethasone. All responses were assessed according to International Myeloma Working Group criteria.

* High-risk cytogenetic abnormalities were defined as gain(1q), t(4;14), t(14;16), t(14;20), and del(17p). Ultra-high risk was defined as the presence of more than one high-risk lesion.

† Stratification factor in minimisation algorithm.

**Figure 2:**
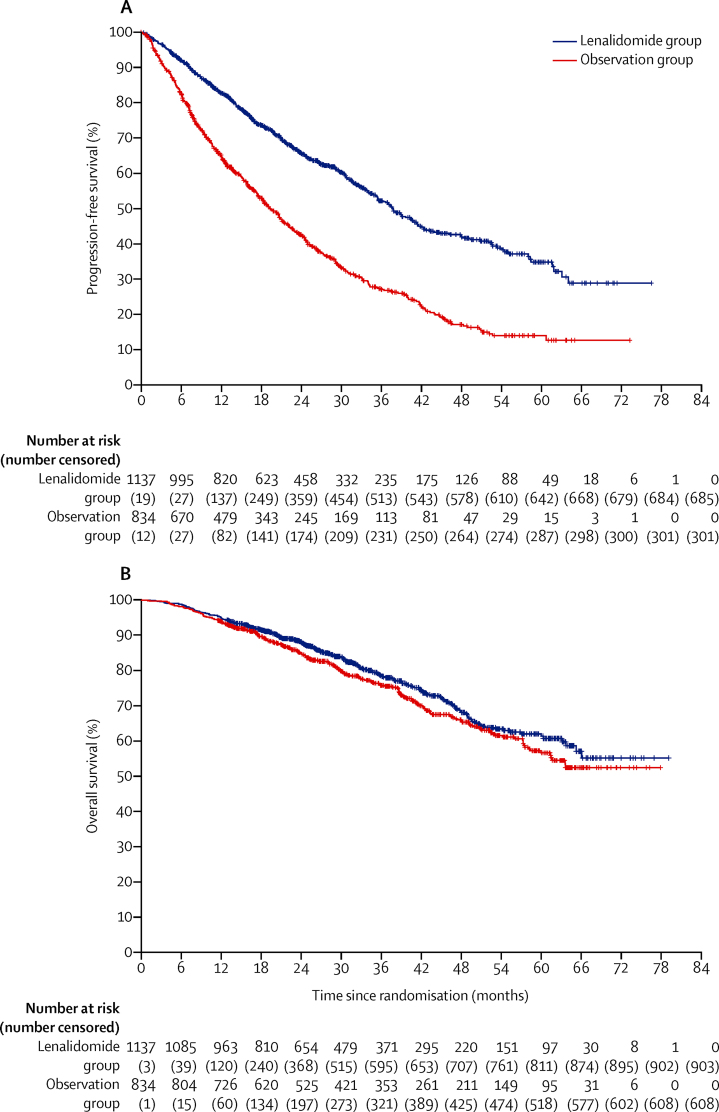
Kaplan-Meier plots of progression-free survival (A) and overall survival (B) in the intention-to-treat population

The median follow-up after randomisation for this analysis was 31 months (IQR 18–50). For the primary analyses, 456 (40%) of 1137 patients in the lenalidomide group and 533 (64%) of 834 patients in the observation group had disease progression or died. Median progression-free survival was 39 months (95% CI 36–42) with lenalidomide and 20 months (18–22) with observation (HR 0·46 [95% CI 0·41–0·53]; p<0·0001; figure 2A). A sensitivity analysis including those patients who had disease progression before maintenance randomisation, in which events were defined at the time of maintenance randomisation rather than being censored at the time of maintenance randomisation, gave similar results (appendix p 33).

234 (21%) of 1137 patients died in the lenalidomide group and 226 (27%) of 834 patients died in the observation group. Median overall survival was not reached (95% CI 66–not reached) with lenalidomide and not reached (61–not reached) with observation. 3-year overall survival was 78·6% (95% Cl 75·6–81·6) in the lenalidomide group and 75·8% (72·4–79·2) in the observation group, and 5-year overall survival was 61·3% (95% Cl 56·6–66·1) in the lenalidomide group and 56·6% (51·5–61·7) in the observation group. No difference was detected between lenalidomide and observation for overall survival (HR 0·87 [95% CI 0·73–1·05]; p=0·15; figure 2B). The most common cause of death was tumour load (appendix p 25).

**Figure 3:**
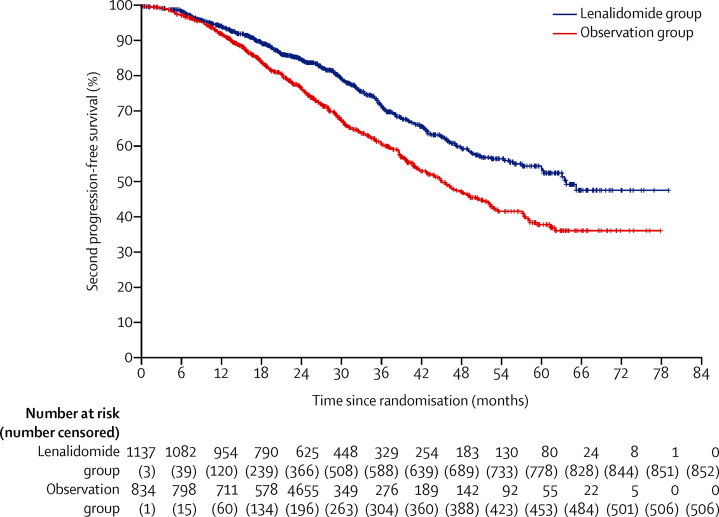
Kaplan-Meier plot of progression-free survival 2 in the intention-to-treat population

285 (25%) of 1137 patients in the lenalidomide group and 328 (39%) of 834 patients in the observation group had second disease progression or died. Median progression-free survival 2 was 64 months (95% CI 57–not reached) with lenalidomide and 45 months (41–50) with observation (HR 0·65 [95% CI 0·56–0·77]; p<0·0001; figure 3).

At the time of analysis, the median duration of lenalidomide maintenance therapy was 18 cycles (IQR 6–30). Dose modifications were applied to 781 (69%) of 1137 patients allocated to lenalidomide treatment. Reasons for discontinuing in the 594 patients who had ceased lenalidomide maintenance were disease progression or death in 320 (54%) patients, adverse events in 167 (28%) patients, patient preference in 45 (8%) patients, other reasons for 43 (7%) patients, and unknown reasons for 19 (3%) patients. Adverse events were assessed in the 1097 patients who completed at least one dose of study drug (table 2). The most common grade 3 or 4 haematological adverse reactions in the lenalidomide group were neutropenia in 362 (33%) patients, thrombocytopenia in 72 (7%) patients, and anaemia in 42 (4%) patients. Serious adverse events were reported in 494 (45%) of 1097 patients receiving lenalidomide compared with 150 (17%) of 874 patients on observation (appendix p 29). The 3-year cumulative incidence of second primary malignancies was low, but higher in the lenalidomide group than the observation group (5·3% [95% CI 3·6–7·1] *vs* 3·1% [1·8–4·5]; HR 1·85 [95% CI 1·18–2·90]; appendix p 5). The overall incidence of second primary malignancies per 100 patient-years was 2·4 (95% CI 1·9–3·1) in the lenalidomide group and 1·4 (1·0–2·0) in the observation group. The 3-year cumulative incidence of deaths related to second primary malignancies was low in both groups (2·0% [95% CI 0·9–3·1] in the lenalidomide group *vs* 0·9% [0·2–1·6] in the observation group; appendix p 6). A summary of all second primary malignancies by intervention group is shown in the appendix (p 30).

**Table 2:** Adverse events in patients treated with lenalidomide maintenance therapy (n=1097)

		**Grade 1 or 2**	**Grade 3**	**Grade 4**	**Grade 5**
Haematological					
	Neutropenia	419 (38%)	308 (28%)	54 (5%)	0
	Anaemia	657 (60%)	40 (4%)	2 (<1%)	0
	Thrombocytopenia	489 (45%)	49 (4%)	23 (2%)	0
Infections					
	Lower or upper respiratory infection	261 (24%)	89 (8%)	4 (<1%)	4 (<1%)
	Sepsis	1 (<1%)	12 (1%)	6 (1%)	2 (<1%)
	Other infections and infestations	104 (9%)	23 (2%)	0	0
Neurological					
	Peripheral sensory neuropathy	319 (29%)	9 (1%)	0	0
Gastroenterological					
	Constipation	312 (28%)	1 (<1%)	0	0
	Nausea	140 (13%)	2 (<1%)	0	0
Other					
	Fatigue or lethargy	363 (33%)	15 (1%)	0	0
	Back pain	171 (16%)	5 (<1%)	0	0
	Rash	155 (14%)	8 (1%)	1 (<1%)	0
	Cough	137 (13%)	3 (<1%)	0	0
	Myalgia	128 (12%)	0	2 (<1%)	0
	Arthralgia	115 (10%)	5 (<1%)	0	0
	Cardiac disorder	0	0	0	1 (<1%)

Data are n (%). The table includes grade 1 or 2 adverse events occurring in at least 10% of patients and grade 3 or 4 events in at least 1% of patients (the rest of the grade 3 and 4 adverse events are in the appendix pp 26–28). All grade 5 events are shown. As per protocol, only serious adverse events were recorded in the observation group, and are presented in the appendix (p 29).

The most common serious adverse events were infections in both the lenalidomide group and the observation group. 460 deaths occurred during maintenance treatment, 234 (21%) in the lenalidomide group and 226 (27%) in the observation group, and no deaths in the lenalidomide group were reported as treatment-related (appendix p 25).

**Figure 4:**
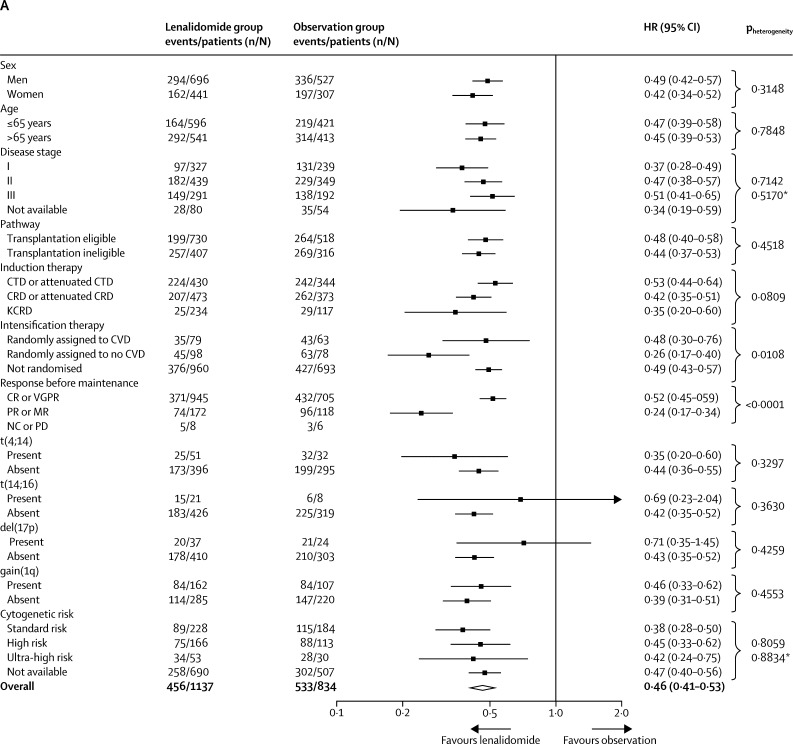

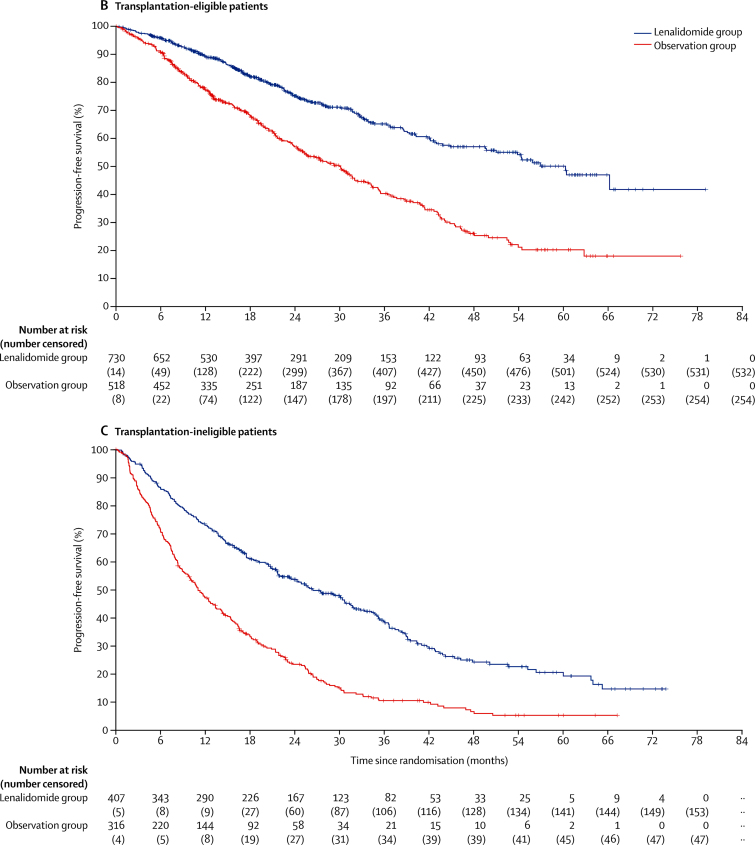
Subgroup analysis of progression-free survival (A) Forest plot of progression-free survival in the intention-to-treat population. For the response before maintenance (NC or PD) subgroup, the Cox model was inestimable because of the small numbers of patients and events in the subgroup. Comparisons by sex, International Staging System disease stage, and response before maintenance were done as post-hoc analyses. The test for heterogeneity in the Response before maintenance setting only applied to the CR or VGPR and PR or MR subgroups. (B) Kaplan-Meier plot of progression-free survival in transplantation-eligible patients. (C) Kaplan-Meier plot of progression-free survival in transplantation-ineligible patients. CR=complete response. CRD=cyclophosphamide, lenalidomide, and dexamethasone. CTD=cyclophosphamide, thalidomide, and dexamethasone. CVD=cyclophosphamide, bortezomib, and dexamethasone. HR=hazard ratio. KCRD=carfilzomib, cyclophosphamide, lenalidomide, and dexamethasone. MR=minimal response. NC=no change. PD=progressive disease. PR=partial response. VGPR=very good partial response. *Likelihood ratio test for heterogeneity of effect among patients with subgroup data available.

In the subgroup analyses, the benefit of lenalidomide on progression-free survival was seen across most subgroups of patients (figure 4A), including those prespecified (transplantation-eligible and transplantation-ineligible patients and those defined as standard risk, high-risk and ultra-high risk genetics). The only significant heterogeneity was between patients who achieved a complete or very good partial response and those who had a partial or minimal response before maintenance therapy (p_heterogeneity_<0·0001). Although progression-free survival improved significantly with lenalidomide maintenance in both responders and non-responders, patients with a suboptimal response before maintenance therapy appeared to benefit the most.

In an analysis by transplantation eligibility status, which was pre-specified in the statistical plan, median progression-free survival was 57 months (95% CI 50–not reached) in the lenalidomide group and 30 months (25–32) in the observation group in transplantation-eligible patients (HR 0·48 [95% CI 0·40–0·58]; p<0·0001; figure 4B), and in transplantation-ineligible patients, median progression-free survival was 26 months (95% CI 22–31) with lenalidomide and 11 months (5–23) with observation (HR 0·44 [95% CI 0·37–0·53]; p<0·0001; figure 4C).

For the prespecified subgroup analysis by cytogenetic risk group, the baseline characteristics between patients with (774 [39%] of 1971) and without (1197 [61%] of 1971) cytogenetic data available were balanced (appendix p 31). Progression-free survival was improved in patients of all cytogenetic risk groups (standard risk, high risk, and ultra-high risk) who received lenalidomide compared with those on observation (appendix p 9); the benefit of lenalidomide was also seen across these risk categories when the intention-to-treat population was divided into transplantation-eligible and transplantation-ineligible patients, with no significant heterogeneity between genetic subgroups (p_heterogeneity_=0·98 for transplantation-eligible patients, p_heterogeneity_=0·76 for transplantation-ineligible patients; appendix pp 10–11). Similar results for progression-free survival were seen if the definition of high risk was restricted to patients with t(4;14), t(4;14) and del(17p), or del(17p) compared with those without either t(4;14) or del(17p), independently of transplantation eligibility (appendix pp 12–14). Further subgroup analyses of progression-free survival by induction treatment and transplantation status also showed a benefit for lenalidomide maintenance compared with observation regardless of sex, age, disease stage, and response at baseline (appendix pp 7–8).

**Figure 5:**
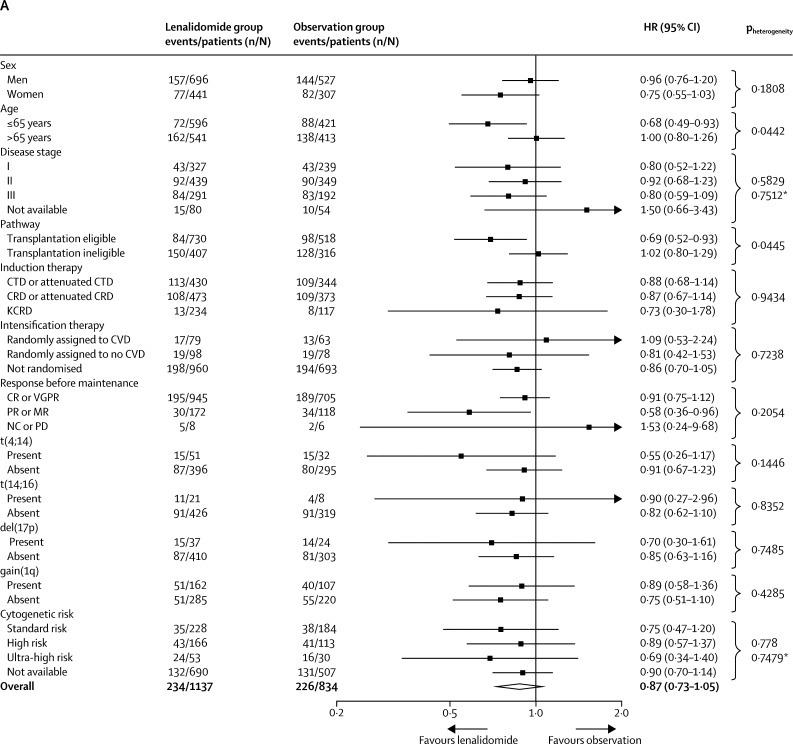

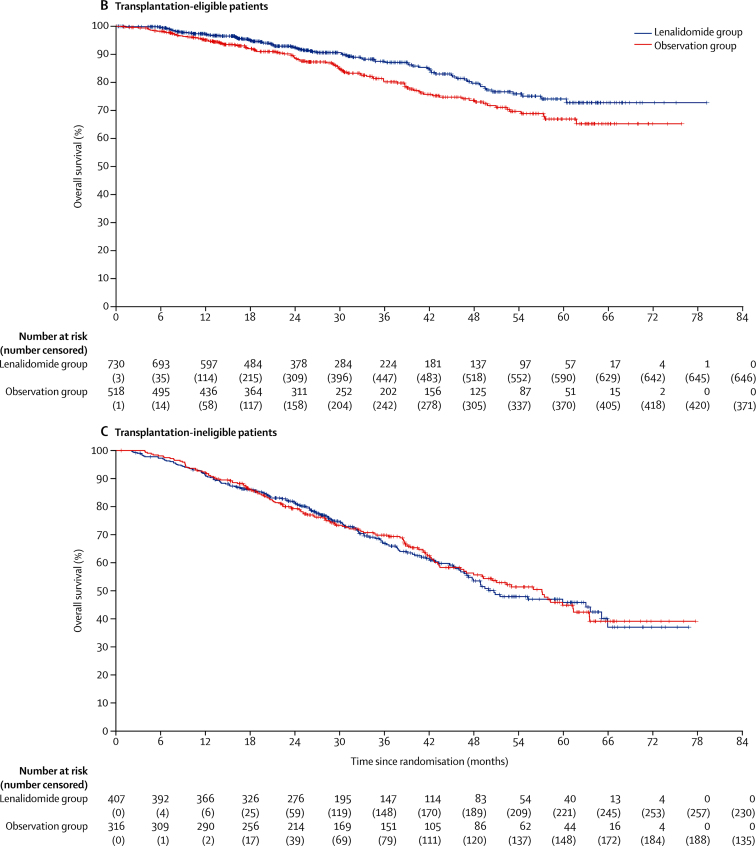
Subgroup analysis of overall survival (A) Forest plot of overall survival in the intention-to-treat population. (B) Kaplan-Meier plot of overall survival in transplantation-eligible patients. (C) Kaplan-Meier plot of overall survival in transplantation-ineligible patients. CR=complete response. CRD=cyclophosphamide, lenalidomide, and dexamethasone. CTD=cyclophosphamide, thalidomide, and dexamethasone. CVD=cyclophosphamide, bortezomib and dexamethasone. HR=hazard ratio. KCRD=carfilzomib, cyclophosphamide, lenalidomide, and dexamethasone. MR=minimal response. NC=no change. PD=progressive disease. PR=partial response. VGPR=very good partial response. *Likelihood ratio test for heterogeneity of effect amongst patients with subgroup data available.

In the subgroup analysis of overall survival (figure 5A), there was significant heterogeneity in outcomes based on pathway for previous induction treatment (p_heterogeneity_=0·0445) and age (p_heterogeneity_=0·0442), with age probably acting as a surrogate for transplantation pathway eligibility. A significant improvement in overall survival was seen in transplantation-eligible patients treated with lenalidomide compared with those assigned to observation (3-year overall survival in transplant-eligible patients 87·5% [95% Cl 84·3–90·7] with lenalidomide and 80·2% [76·0–84·4] with observation; HR 0·69 [95% CI 0·52–0·93]; p=0·014; figure 5B). However, lenalidomide maintenance therapy did not improve overall survival in transplantation-ineligible patients (3-year overall survival 66·8% [61·6–72·1] with lenalidomide and 69·8% [64·4–75·2] with observation; HR 1·02 [95% CI 0·80–1·29]; p=0·88; figure 5C). The benefit of lenalidomide maintenance therapy on overall survival in the transplantation-eligible patients was also confirmed across subgroups based on age, disease stage, induction therapy, and response at baseline (appendix p 15). Heterogeneity was detected in overall survival benefit by sex (appendix p 15). No subgroup appeared to benefit from lenalidomide maintenance in the transplantation-ineligible pathway (appendix pp 15–16). In the subgroup analysis of overall survival by transplantation status, there was no heterogeneity between cytogenetic risk groups (appendix pp 15–22). By cytogenetic risk group, in standard-risk patients, 3-year overall survival was 86·4% (95% CI 80·0–90·9) in the lenalidomide group compared with 81·3% (74·2–86·7] in the observation group, and in high-risk patients, it was 74·9% (65·8–81·9) in the lenalidomide group compared with 63·7% (52·8–72·7) in the observation group; and in ultra-high-risk patients it was 62·9% (46·0–75·8) compared with 43·5% (22·2–63·1).

**Figure 6:**
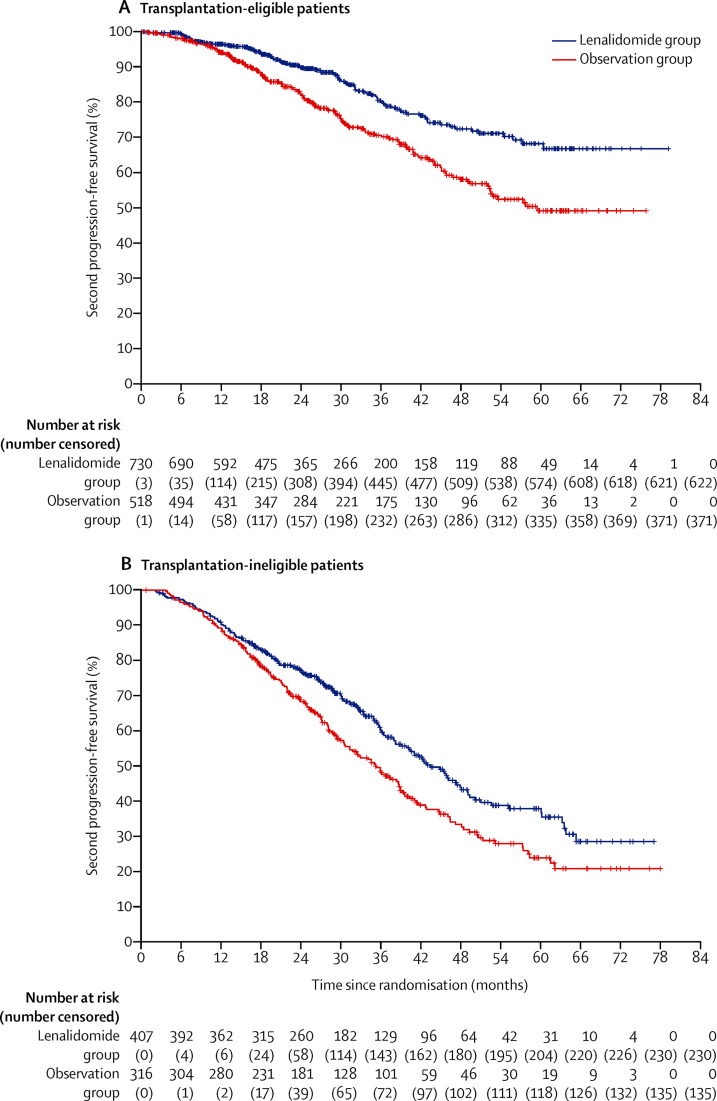
Kaplan-Meier plot of progression-free survival 2 in transplantation-eligible patients (A) and transplantation-ineligible patients (B)

A significant improvement in progression-free survival 2 was seen with lenalidomide maintenance in transplantation-eligible patients (figure 6A) and in transplantation-ineligible patients (figure 6B). In transplantation-eligible patients, median progression-free survival was not reached for the lenalidomide group and 59 months (95% CI 52–not reached) for the observation group (HR 0·57, 95% CI 0·44–0·73; p<0·0001); and for transplant-ineligible patients it was 43 months (39–48) for the lenalidomide group and 35 months (31–39) for the observation group (HR 0·72, 0·58–0·88; p=0·0016). A post-hoc analysis of progression-free survival 2 with lenalidomide compared with observation in different patient groups is shown in appendix p 23).

Because of the apparent benefit of lenalidomide maintenance on progression-free survival 2, we did some post-hoc exploratory analyses looking at factors that might have affected overall survival outcomes, particularly in the transplantation-ineligible group. The transplantation-ineligible group of patients had a higher frequency of non-myeloma-related deaths (appendix p 25), and a high proportion of these patients initially assigned to the observation group subsequently received lenalidomide (103 [33%] of 316 patients) or ceased treatment before disease progression without intolerance (119 [29%] of 407 patients).

Finally, to take account of all available results of lenalidomide as maintenance therapy for patients with multiple myeloma, we did a summary meta-analysis of our data with those previously published, including 3179 patients. These results show the HR for overall survival with the use of a lenalidomide maintenance regimen was 0·72 (95% CI 0·56–0·91; appendix p 24).

The heterogeneity statistic *I*^2^=54·6 indicated potential moderate heterogeneity. However, this heterogeneity could be considered of questionable importance given that all studies showed consistent treatment effects in favour of lenalidomide. No analysis of risk of bias was undertaken.

## Discussion

In this phase 3, open-label, randomised trial—which is, to our knowledge, the largest of its kind—lenalidomide maintenance significantly improved progression-free survival compared with observation in adult patients with newly diagnosed multiple myeloma. However, overall survival was not improved with this treatment regimen in the intention-to-treat analysis.

The progression-free survival benefit of maintenance therapy with lenalidomide seems to continue through the subsequent line of therapy with progression-free survival 2 significantly improved with lenalidomide compared with observation in both transplantation-eligible and transplantation-ineligible patients. This finding suggests that lenalidomide maintenance might not negatively affect the activity of subsequent treatment by selecting for more aggressive or drug-resistant myeloma clones. This hypothesis is also supported by previous analyses indicating that lenalidomide maintenance did not increase genomic change or mutational load.^26^, ^27^

A prespecified subgroup analysis suggested that continuous lenalidomide improved overall survival in transplantation-eligible patients but not in transplantation-ineligible patients. We speculate that any overall survival benefit in transplantation-ineligible patients might have been attenuated by a high proportion of patients ceasing treatment prematurely and those initially randomised to observation who received lenalidomide in later lines of treatment. Long-term follow-up of this study is planned and will specifically examine the effect of these factors on outcomes. Additional secondary and exploratory endpoints including response, survival after progression, time to progression, time to next line of treatment, changes in mean paraprotein during protocol treatment, and time to improved response will also be presented subsequently.

An adverse cytogenetic profile is a major risk factor for relapse, and outcomes for patients with high-risk cytogenetics are poor.^1^, ^3^, ^28^ Data supporting the use of lenalidomide maintenance in patients with adverse cytogenetics are scarce, and results thus far have been inconclusive. In the IFM-2005-02 trial,^7^ lenalidomide maintenance therapy improved median progression-free survival in patients with del(17p) compared with observation (29 months *vs* 14 months), whereas those with t(4;14) did not have a benefit (28 months *vs* 24 months), probably because of the small sample size.

Our study has limitations. Although the Myeloma XI had a much larger sample size than that of previous studies, our subgroup analyses were not powered and therefore are not conclusive and warrant further investigation. Additionally, cytogenetic data were not available for all patients, further reducing the sample size, and should be interpreted with caution. However, baseline characteristics and outcomes were similar between patients with and without cytogenetic data available.

Another limitation of the trial is the lack of prospectively collected quality-of-life data. However, given that the time until first disease progression is often when patients with myeloma have a better quality of life, the improvement in progression-free survival and progression-free survival 2 seen in our study suggests the use of maintenance lenalidomide would be of benefit in the transplantation-ineligible setting.

Cytogenetic high risk has been defined by several groups using different markers. In our previous study,^3^ we identified adverse cytogenetic abnormalities as gain(1q), t(4;14), t(14;16), t(14;20), or del(17p), with standard risk (no adverse cytogenetic abnormalities); high risk (one adverse cytogenetic abnormality), or ultra-high risk (two or more adverse cytogenetic abnormalities) groups identified, and this classification was used in our prespecified analysis. Other groups limit the high-risk classification to patients with t(4;14) or del(17p). The size of the Myeloma XI study has enabled us to examine the data using both of these classification systems. Nevertheless, lenalidomide maintenance cannot equilibrate the outcomes for high-risk patients to those of standard risk, and patients with high-risk lesions still have adverse outcomes. Strategies combining maintenance lenalidomide with additional agents might be beneficial and could be investigated. In the Myeloma XI trial, a separate group of patients received vorinostat plus lenalidomide in the maintenance randomisation because of a protocol amendment, and the outcomes of these patients will be reported elsewhere. Other potential combinations would be to combine maintenance lenalidomide with a well tolerated proteasome inhibitor or CD38 antibody.

The results of this trial are consistent with previous findings from phase 3 trials of lenalidomide maintenance therapy. In transplantation-eligible patients, three studies have shown that the addition of lenalidomide maintenance after autologous stem-cell transplantation reduces the risk of progression or death by approximately 50% compared with placebo or no maintenance.^7^, ^8^, ^9^ A significant improvement in overall survival has only been observed in one previous trial,^8^ although a recent meta-analysis did suggest an overall survival improvement in patients treated with lenalidomide in this setting.^15^ To take account of all available results so far now including the Myeloma XI study, we did a summary data meta-analysis, which showed that lenalidomide maintenance therapy reduced the risk of death.

For transplantation-ineligible patients with multiple myeloma, a significant improvement in progression-free survival has been reported when lenalidomide maintenance was given after a regimen of melphalan, prednisone, and lenalidomide (median progression-free survival from start of induction 31 months *vs* 14 months); the 3-year overall survival in this study was 70% with maintenance and 62% without maintenance therapy.^29^ Furthermore, in a study comparing treatment with lenalidomide and low-dose dexamethasone given either continuously or for a limited number of cycles,^21^ continuous treatment significantly improved progression-free survival (median 26 months *vs* 21 months from start of induction, HR 0·70, 95% CI 0·60–0·81) but there was no difference in overall survival (median 59·1 months *vs* 62·3 months, 1·02, 0·86–1·20). Taken together, these observations suggest that alternative approaches to improving overall survival in older patients who are not eligible for transplantation are warranted.

The current findings with lenalidomide maintenance compare favourably to those achieved with other novel therapies in the maintenance setting. Thalidomide has been shown to improve progression-free survival when given as maintenance therapy in the Myeloma IX trial (median progression-free survival 22 months *vs* 15 months; HR 1·44, 95% CI 1·22–1·70; p<0·0001), but the median overall survival was similar in both groups (60 months *vs* 60 months; 0·96, 0·79–1·17; p=0·70).^10^, ^30^ In a subgroup analysis, patients with high-risk status receiving thalidomide had no progression-free survival or overall survival benefit. By contrast, in this current study, we have shown that lenalidomide improves progression-free survival irrespective of cytogenetic risk status, which might be due to mechanistic differences between these drugs. Although thalidomide and lenalidomide share a similar mechanism of action, recent studies suggest that subtle variations in the chemical structure between the molecules can modulate the range of substrates targeted for degradation by the E3 ubiquitin ligase, leading to different downstream effects.^31^ In the Myeloma XI trial, few patients discontinued because of adverse events or patient preference and there was no evidence of cumulative toxicity. The risk of second primary malignancies was increased in patients treated with maintenance lenalidomide, but the 3-year cumulative incidence of second primary malignancies remained low in both treatment groups.

In summary, lenalidomide maintenance therapy significantly improved progression-free survival in patients with newly diagnosed multiple myeloma compared with observation, but overall survival was not improved in the intention-to-treat analysis across the whole trial population. Additionally, prespecified subgroups analyses by cytogenetic risk and transplantation status suggested a progression-free survival benefit across all cytogenetic risk groups, and an overall survival benefit in transplantation-eligible patients.

## Data sharing
